# Emergent multisystemic *Enterococcus* infection threatens endangered Christmas Island reptile populations

**DOI:** 10.1371/journal.pone.0181240

**Published:** 2017-07-20

**Authors:** Karrie Rose, Jessica Agius, Jane Hall, Paul Thompson, John-Sebastian Eden, Mukesh Srivastava, Brendan Tiernan, Cheryl Jenkins, David Phalen

**Affiliations:** 1 Taronga Conservation Society Australia, Sydney, New South Wales, Australia; 2 College of Public Health, Medical and Veterinary Sciences, James Cook University, Townsville, Queensland, Australia; 3 Faculty of Veterinary Science, Sydney School of Veterinary Science, University of Sydney, Sydney, New South Wales, Australia; 4 Marie Bashir Institute for Infectious Diseases and Biosecurity, Charles Perkins Centre, School of Life and Environmental Sciences and Sydney Medical School, the University of Sydney, Sydney, New South Wales, Australia; 5 Elizabeth Macarthur Agricultural Institute, New South Wales Department of Primary Industries, Camden, New South Wales, Australia; 6 Christmas Island National Park, Drumsite, Territory of Christmas Island, Australia; Charles University, CZECH REPUBLIC

## Abstract

Multisystemic infections with a morphologically unusual bacterium were first observed in captive critically endangered Lister’s geckos (*Lepidodactylus listeri*) on Christmas Island in October 2014. Since then the infection was identified in another captive critically endangered lizard species, the blue-tailed skink (*Cryptoblepharus egeriae*) and two species of invasive geckos; the four clawed gecko (*Gehyra mutilata*) and Asian house gecko (*Hemidactylus frenatus*), in a wide geographic range across the east side of the island. The Gram and periodic acid-Schiff positive cocci to diplococci have a propensity to form chains surrounded by a matrix, which ultrastructurally appears to be formed by fibrillar capsular projections. The bacterium was associated with severe and extensive replacement of tissues, but minimal host inflammatory response. Attempts to grow the organism in culture and in embryonated eggs were unsuccessful. Molecular characterisation of the organism placed it as a novel member of the genus *Enterococcus*. Disease Risk Analyses including this organism should now be factored into conservation management actions and island biosecurity.

## Introduction

Christmas Island (-10.412428 to -10.571063, 105.533096 to 105.712163) is a remote equatorial island territory of Australia located in the Indian Ocean that supports a diverse ecosystem rich in endemic species of great conservation and scientific value [1, 2]. Since its discovery, however, human activity is believed to have resulted in declines and extinctions of mammals, birds, and reptiles on the island [1]. Of the five native reptiles on Christmas Island, four species including the coastal skink (*Emoia astrocostata*) and the endemic Lister’s gecko (*Lepidodactylus listeri*), blue-tailed skink (*Cryptoblepharus egeriae*), and the forest skink (*Emoia nativitatis*) are either extinct in the wild, or are persisting at very low levels [1, 3–5]. Only the Christmas Island giant gecko (*Cyrtodactylus sadleiri*) remains relatively abundant, although its population has reportedly contracted since surveys in 1979 and the 1990s [5, 6], and little is known about the conservation status of the rarely recorded Christmas Island blind snake (*Ramphotyphlops exocoeti*). The Lister’s gecko and blue-tailed skink are conservation dependent, surviving only in captive breeding programs on Christmas Island, and at Taronga Zoo, Sydney, Australia. The precise cause of the declines of these reptile populations is not known. However, numerous anthropogenic processes on Christmas Island, including phosphate mining, land clearing, rainforest fragmentation, the emission of potentially harmful pollutants, and the introduction of a vast array of invasive species, are suspected to be contributing factors, either individually or in combination [1, 5]. The contribution of introduced or emerging infectious diseases to Christmas Island reptile population declines is uncertain, but an introduced pathogen has been implicated in the extinction of the endemic Maclear’s rat (*Rattus macleari*) and bulldog rat (*Rattus nativitatis*) [7].

Diseases of infectious origin have been increasingly recognised as impacting reptiles both locally and on a global scale [8]. Examples of infectious diseases affecting reptile communities include herpesvirus-induced fibropapillomatosis in marine turtles [9] and yellow fungus disease in an array of reptile species [10]. In general, bacterial infections in reptiles affect only individual animals [11, 12], occasionally, however, they can have local and population-wide impacts. An outbreak of *Mycoplasma agassizii* and *Mycoplasma testudineum*, has caused extensive upper respiratory tract disease and widespread morbidity and mortality events in Agassiz’s desert tortoise (*Gopherus agassizii*) [13]. Additionally, infections caused by a number of *Enterococcus* species, predominantly *Enterococcus faecium* and *Enterococcus faecalis*, caused significant disease in 50 hospitalised Kemp’s Ridley sea turtles (*Lepidochelys kempii*), with affected animals presenting with bacteraemia, septicaemia and necrotising osteomyelitis [14].

Enterococci are ubiquitous [15] and commonly isolated from an array of environmental niches as a result of their ability to survive an extensive range of environmental conditions [16] and adapt to a variety of stressors otherwise considered extremely hostile for other bacterial species [15,17]. Enterococci form part of the commensal gastrointestinal microbiota of a diverse range of taxa, inclusive of mammals, birds, reptiles and insects. These organisms are commonly isolated from surface water, soil, waste and raw animal and plant products [18]. Although largely considered commensals, enterococci are opportunistic and have progressively been recognised as pathogenic agents of disease in both humans and animals [19–21]. Microbial infection of reptilian fauna with species from the *Enterococcus* genus can be commensal or cause a continuum of clinical manifestations including lethargy, bacteraemia, septicaemia, necrotising osteomyelitis and death [14, 22–25].

In this study we investigate the cause of facial deformity and multisystemic bacterial infection in native and invasive reptiles on Christmas Island, which has implications for conservation management, species survival, and biosecurity.

## Materials and methods

### Animal Ethics

Euthanasia of sick reptiles was undertaken when they were deemed to be severely ill and losing body condition. These animals were euthanased by placing them in a sterile, air-tight container with an isoflurane (Pharmachem, Eagle Farm, QLD, Australia) soaked cotton ball, resulting in deep anaesthesia and ultimately death.

All live wild reptiles were collected using procedures approved by the Taronga Zoo Animal Ethics Committee (approval no. 4b/05/10) in compliance with the Australian Code of Practice for the Use of Animals for Scientific Purposes. Taronga Zoo Animal Ethics committee specifically approved the management and handling of live animals that are included in this study. Tissues from captive native and wild invasive reptiles that died or were euthanased due to the severity of their disease were included in this study under the Taronga Zoo Animal Ethics Committee's policy and procedures for opportunistic sample collection, a program that operates under the auspices of New South Wales Department of Primary Industries.

### Sources of reptiles included in this study

#### Prospective analysis

**Fig 1:**
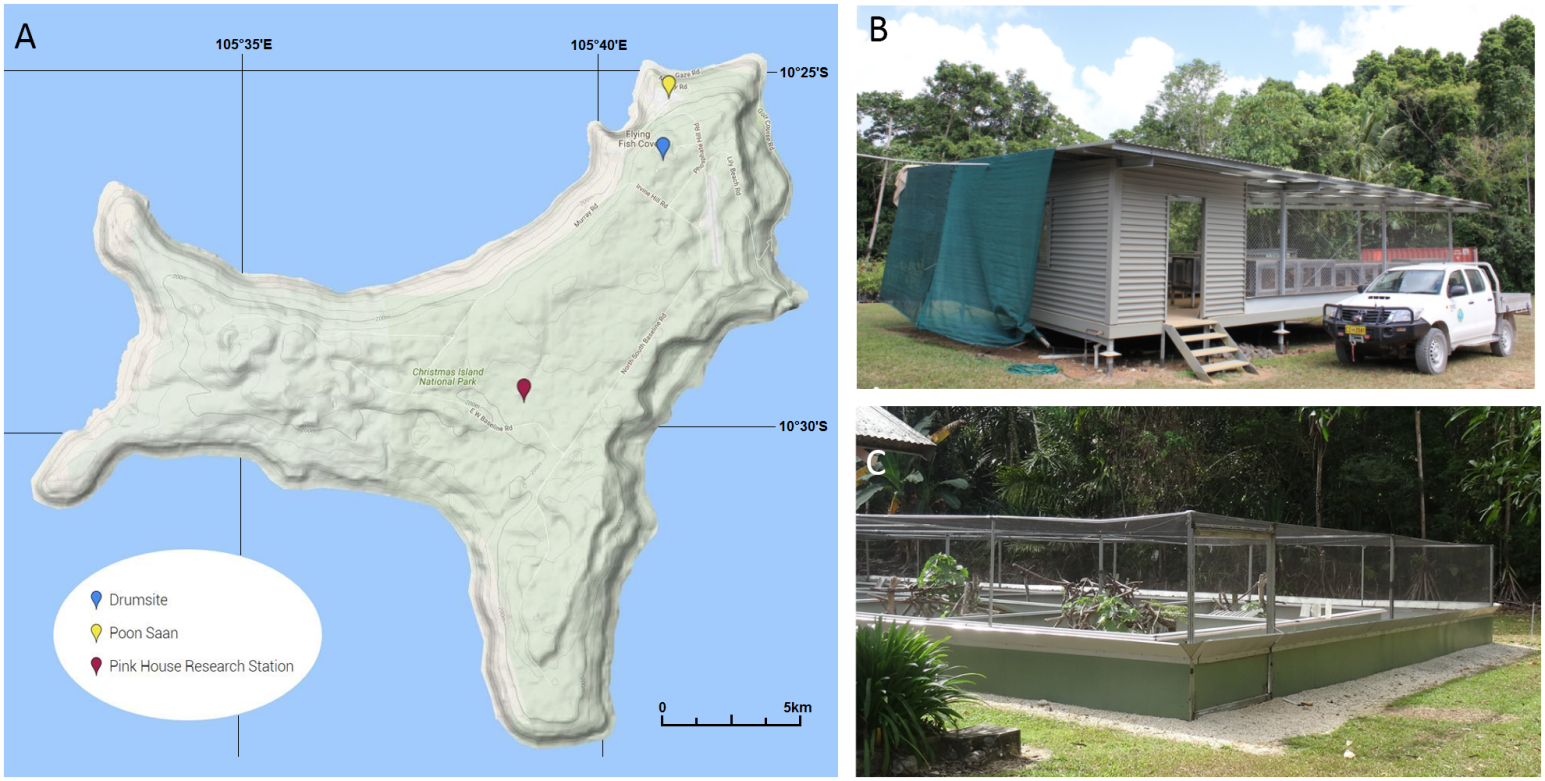
Christmas Island map and reptile breeding facilities. Map of Christmas Island showing the locations where infected animals were observed (A). Covered breeding facility for Lister’s geckos and blue-tailed skinks (B). Enclosed outdoor breeding facility for blue-tailed skinks (C).

The prospective cases in this study came from two sources: 1) diseased, native Lister’s geckos (*L*. *listeri*) (n = 36) and blue-tailed skinks (*C*. *egeriae*) (n = 1) that were housed in the captive breeding colony (Pink House research station) on Christmas Island and 2) diseased, invasive, free-ranging, four-clawed geckos (*Hemidactylus*. *frenatus*) (n = 3) and Asian house geckos (*Gehyra mutilata*) (n = 1) opportunistically collected between March 2015 and September 2016 at three sites (Pink House, Drumsite, and Poon Saan) on Christmas Island (Fig 1A). The captive breeding facility on Christmas Island for Lister’s geckos was a single room that was raised from the ground substrate and enclosed. Three walls were composed of chicken wire mesh (Fig 1B). Lister’s geckos were housed in glass terrariums covered with a plexiglass top. Mesh covered holes in the plexiglass allowed for ventilation and the potential for exposure to feral reptiles. The terrariums contained untreated soil and branches that were obtained from the local environment. Short sections of polyvinyl chloride pipe were used as shelters. Approximately 20 Lister’s geckos were housed per terrarium. Blue-tailed skinks were also housed in this room in identical terrariums. Additional blue-tailed skinks were housed in an adjacent outdoor enclosure composed of a solid wall to 1m high, which was extended in height and covered with mesh (Fig 1C). Smaller solid wall enclosures within this outdoor structure were used to house the blue-tailed skinks. The smaller enclosures were not covered and had dirt floors. Lister’s geckos and blue-tailed skinks were fed crickets bred on the island and insects caught by the animal carers from the local environment. Feral Asian house geckos and four-clawed geckos were routinely seen in both the indoor breeding room and the outdoor enclosures.

#### Retrospective analysis

Data were obtained from three retrospective data sets. The first reptiles examined were whole ethanol-fixed reptile specimens maintained at the Australian Museum that were collected from multiple locations across Christmas Island between May 1979 and June 1998 [6]. They were examined grossly with the aid of magnification for evidence of facial deformities and nodular lesions of the skin that are characteristic of the tissue destruction and bacterial colony formation associated with this disease. Specimens examined included 53 Christmas Island giant geckos, 87 coastal skinks, 36 grass skinks, 11 forest skinks, 53 blue-tailed skinks, 69 Lister’s geckos, 73 Asian house geckos, and 17 four-clawed geckos. The second source of retrospective specimens was collected in 2010 as part of an island-wide health surveillance project [26]. Animals examined were 2 giant geckos, 60 grass skinks, 4 flowerpot blind snakes (*Ramphotyphlops braminus*), 245 Asian house geckos, 50 wolf snakes, and 27 four-clawed geckos. These animals were examined grossly and histologically. Aseptically collected liver samples from almost all of the reptiles collected in 2010 were frozen and transported in liquid nitrogen, then stored at -80°C for ten months prior to being pooled by species in groups of five, and inoculated into VERO and Russell’s viper (*Daboia russelii*) cardiac fibroblast cell lines (American Type Culture Collection, Manassa, VA, USA, cell line CCL-140). These cell lines were incubated at 30°C for 21 days during which time they were subjected to three passages and examined twice weekly for cytopathic effects. Two sets of pooled samples were subject to electron microscopy.

The last sources of retrospective specimens were Lister’s geckos (n = 32) and blue-tailed skinks (n = 96) from the Taronga Zoo, Sydney, Australia captive breeding colony. The captive breeding colony originated from animals imported from Christmas Island in 2009 and 2011. As part of disease surveillance in this collection, all animals that died were examined grossly and histologically. The captive breeding facility at Taronga Zoo was a biosecure room with restricted access containing glass and plastic terraria, substrate and cage furniture that were sterilised before use. Geckos and skinks in this breeding facility were fed insects raised by the Zoo’s staff.

### Sample collection and preservation

Living reptiles were euthanased by placing them in a sterile, air-tight container with an isoflurane (Pharmachem) soaked cotton ball, resulting in deep anaesthesia and ultimately death. A small ventral incision was made using DNA-free instruments, and then a second set of clean instruments were used to remove half of the animal’s liver lobe. Liver samples were preserved in 70% ethanol for DNA extraction. Additional liver samples were placed in the following transport solutions: azide dextrose broth (a solution of sodium azide, glucose, peptone, sodium chloride, di-potassium hydrogen phosphate and potassium di-hydrogen phosphate, (Thermo Scientific, Scoresby, VIC, Australia) stored at 4°C, 10% DMSO in double distilled water or 50% glycerol in double distilled water and frozen in liquid nitrogen vapour. Cadaver tissues were either fixed in 10% neutral buffered formalin or in 70% ethanol. All specimens collected on the island were transported to the Australian Registry of Wildlife Health in Sydney for further analysis.

### Microscopic examination of the tissues from the diseased lizards and morphological characterisation of the bacterium

Smears made from direct impressions of lesions onto glass microscope slides were stained with Romanowsky (Rapid Diff Stain no. 1, Australian Biostains, Tragalgon, VIC, Australia), Gram, and periodic acid Shiff (PAS) stains (Veterinary Pathology Diagnostic Services, University of Sydney, Sydney, NSW, Australia). Formalin-fixed sagittal sections of affected reptiles were paraffin-embedded, sectioned at 4 μm, and stained with hematoxylin and eosin (H&E). Selected sections were also stained with PAS, alcian blue, mucicarmine, Giemsa, and Gram stains (Veterinary Pathology Diagnostic Services).

### Microbiology

Two attempts were made to culture the organisms from affected lizard tissues transported from Christmas Island to mainland Australia. The first attempt was made using aseptically collected frozen tissue stored at -80°C. The second attempt was made after the organism was genetically characterised as an *Enterococcus* species. The second set of tissues were aseptically collected from affected lizards, immediately frozen in 50% glycerol, or 10% DMSO, or inoculated into azide dextrose broth, as recommended by the American Public Health Association for isolation of faecal enterococci [27]. Culture attempts were undertaken using a variety of bacteriological media and incubation conditions, as summarised in Table 1 [28–31]. Additionally, fresh tissue homogenates from an infected Lister’s gecko and Asian house gecko transported from Christmas Island in azide dextrose broth were homogenised and diluted 1:10 in azide dextrose broth (Thermo Scientific). The homogenates were separately inoculated onto 75% confluent Russell’s viper (*Daboia russelii*) cardiac fibroblasts (American Type Culture Collection, Manassa, USA, cell line CCL-140) that were grown and maintained according to the supplier’s recommendations. After a 7 day period of incubation, Gram stains of the supernatant were made and swabs of the fluid were cultured on blood agar.

**Table 1:** Summary of microbial culture methods.

Species*	Inoculum	Media†	Environment	Temperature	Time
*Ll* (2)	Liver homogenate post -80°C storage	HBA, MCA, CA, SBAss	4.5% CO_2_ enriched, aerobic and anaerobic	35°C	17 days
		SA	Aerobic	30°C	42 days
		CA	Aerobic	4°C, Room temperature, 35°C, 40°C	18 days
		SBAss	4.5% CO_2_ enriched	4°C, Room temperature, 40°C	14 days
		CMb, THb/fbs	Aerobic	35°C	28 days
		CA	Microaerophilic	Room temperature, 35°C, 40°C	14 days
	Liver homogenate post -80°C storage in cooked meat transferred to brain/heart infusion broth after 7 days, then after 18 days transferred to isolator tissue lysis tubes	CA/fbs	Aerobic	Room temperature	14 days
		CA	4.5% CO_2_ enriched	35°C	14 days
	Liver homogenate post -80°C storage	Sm [28]	Aerobic	Room temperature, 35°C	
	Liver homogenate post -80°C storage	BHIb	Aerobic	35°C	7 days
*Gm* (1)	Liver homogenate post -80°C storage	HBA, MCA, CA	4.5% CO_2_ enriched, aerobic and anaerobic	35°C	17 days
		SA	Aerobic	30°C	42 days
*Hf* (4) *Gm* (1)	Fresh liver homogenate transported in Azide dextrose broth 72 hours	SBA + pyridoxine HCl SBA + vitamin solution no. 6 [29] Blood agar base + 5% inactivated horse serum + 5% reptile extract + pyridoxine HCl Blood agar base + 5% inactivated horse serum + 5% reptile extract + vitamin solution no. 6 Blood agar base + 10% inactivated horse serum + pyridoxine HCl Blood agar base + 10% inactivated horse serum + vitamin solution no. 6 Blood agar base + 10% horse serum + pyridoxine HCl Blood agar base + 10% horse serum + vitamin solution no. 6 SBA + vitamin solution + Cysteine HCl	Aerobic, anaerobic, CO_2_ enriched atmosphere.	30°C and 37°C Some also under aerobic conditions at room temperature. Tested for satellitism on each of these media under CO_2_ enriched atmosphere at 30°C.	7 days
	Fresh liver homogenate transported in Azide dextrose broth 72 hours	Chicken embryos inoculated with 50 μL of a 1:10 dilution of each sample.		30°C or 37°C.	7 days
	Fresh liver homogenate transported in Azide dextrose broth 72 hours, and homogenates of liver frozen in either 10% DMSO or 50% glycerol during transport	Russell's viper heart cell line grown in Eagles minimal essential medium + essential amino acids + 5% fetal calf serum.	5% CO_2_ inoculated with a dilution series of fresh tissue.	30°C	7 days

*Species: Lister’s gecko, *Lepidodactylus listeri* (*Ll*), Four-clawed gecko, *Gehyra mutilata* (*Gm*), Asian house gecko, *Hemidactylus frenatus* (*Hf*).

The number of cases for each species are shown in brackets next to the species abbreviation.

^†^Media (Thermo Scientific, Scoresby, VIC, Australia): Horse blood agar (HBA), MacConkey’s agar (MCA), Chocolate agar (CA), Chocolate agar with and without 10% fetal bovine serum (CA/fbs), sheep blood agar (SBA) with Staphylococcal streak (SBAss), Sabauraud’s agar (SA), Cooked meat enrichment broth (CMb), Brain heart infusion broth (BHIb), Thioglycollate broth with and without 10% fetal bovine serum (THb/fbs), Synthetic medium (Sm).

Anaerobic conditions: <1% O_2_, CO_2_ (Oxoid^™^, AnaeroGen^™^, Thermo Scientific).

Homogenates of liver tissue (0.1 mls of a 1:10 dilution of fresh material transported in azide dextrose broth) from four severely affected Asian house geckos and one four-clawed gecko with focal head lesions were each separately injected intravenously into the allantoic vessels of two specific pathogen-free eleven day-old embryonated chicken eggs. Two eggs injected intravenously with phosphate buffered saline were used as controls. Half of the eggs were incubated at 30°C and half at 35°C. All eggs were incubated for 7 days at which time the liver and chorioallantoic membranes (CAMs) were collected. Impression smears of the liver and CAMs were stained with a Gram stain and examined for evidence of bacterial infection. Sections of liver and CAMs where formalin-fixed, paraffin-embedded, sectioned at 4 μm and stained with Gram stain (Veterinary Pathology Diagnostic Services).

### Genetic characterization of the bacterium

#### Sanger sequencing

To identify the bacterium causing these lesions, DNA from an alcohol-fixed, diseased liver from the Lister’s gecko index case was extracted and digested using the Bioline Isolate II Genomic DNA Kit standard protocol (Bioline Pty. Ltd., Alexandria, NSW, Australia). PCR was performed to amplify a variable region of the 16S rDNA using pan bacterial primers DG74 (5'-AGGAGGTGATCCAACCGCA-3') and RW01 (5'-AACTGGAGGAAGGTGGGGAT-3') [32]. Water was used as a negative control and *Escherichia coli* DNA was used as a positive control. Amplification products were separated on a 2% agarose gel containing ethidium bromide and visualised with UV light. The purified amplicons were sequenced in both the forward and reverse direction at the Australian Genome Research Facility (Westmead, NSW, Australia). The sequences were aligned using the CLC Genomics Workbench (https://www.qiagenbioinformatics.com) and a consensus sequence generated (GenBank accession number KX962174.1). The sequence was compared to other known DNA sequences using blastN (National Center for Biotechnology Information, Bethesda MD, USA) [33].

#### Whole genome sequencing

Tissue samples containing bacterial genomic DNA were taken from the head (n = 1) and liver (n = 1) of two grossly and histologically affected ethanol-fixed *H*. *frenatus* specimens collected from Christmas Island during June 2016, and extracted using the Bioline Isolate II Genomic DNA kit standard protocol. The purified DNA from each sample were prepared as shotgun libraries using the Truseq DNA PCR-free library (average insert size of ~350bp), and then sequenced on the Illumina HiSeq X Ten platform producing ~500 million 150bp paired-end reads per DNA library (Macrogen Inc., Seoul, Rep. of Korea). Both samples met the quality control (QC) criteria for sequencing (concentration >20ng/μl, purity (A260/280) >1.7, volume >50μl, and total amount >1μg).

#### Whole genome sequence analysis

The Illumina sequence reads from the head and liver DNA libraries were imported into CLC Genomics Workbench 10 (Qiagen Pty. Ltd., Chadstone, VIC, Australia) and trimmed for quality. The leading ten base pairs from the 5’ end were removed along with ambiguous bases and those with QC scores <15 and sequence reads <20nt. To eliminate host sequences, the trimmed reads were mapped to the reference assembly *Gekko japonicus* V1.1 (GCF_001447785.1) without masking and default mapping parameters. All host mapped reads were then discarded, and the remaining unmapped reads from both libraries were combined and assembled *de novo* using default parameters, with the minimum contig length set to 200 base pairs. This resulted in 3,534,933 contigs with an average length of 507nt. The contig list was then exported and annotated using BLAST [33].

#### Sequence annotation

We employed sequential blasting and filtering to identify and annotate *Enterococcus* contigs from the *de novo* assembled data. Our initial 16S sequencing suggested sequence similarity with *Enterococcus faecium*, therefore, we first performed a screen of the full contig list against a database built from the complete genome of *E*. *faecium* AUS0004 (NC 017022.1) using blastN with an E-value cut-off of 1E-10. To then remove any non-specific hits, we performed a blastN search using the resultant “*Enterococcus faecium*” contig list (569 sequences) against the full non-redundant NCBI nucleotide database with an E-value cut off of 1E-10. Any host or other bacterial hits were removed by filtering only those with highest identify to the genus *Enterococcus*. This final contig list (n = 506) was then annotated with searches against the non-redundant NCBI protein and conserved domain databases using blastX and rpstblast, respectively. From this, genes were identified for use in a multi-locus phylogenetic analysis.

#### Phylogenetic analyses

A total of four constitutive genes with suitable genetic variation [34], including atpA (GenBank accession number MF196190), gdh (GenBank accession number MF196191), gyd (GenBank accession number MF196192) and pstC (GenBank accession number MF196193) were identified and selected from the annotated *Enterococcus* contig list. These sequences were aligned against 12 reference sequences of known *Enterococcus* and outgroup bacteria from the NCBI database. The reference sequences selected for the phylogram comprised a suitable representation of known enterococcal diversity (species), and from which the same constitutive genes (atpA, gdh, gyd and pstC) could be identified. Nucleotide sequence alignments were made individually for each of the genes (atpA, gdh, gyd, pstC) with MEGA6 [35] using the CLUSTALW alignment method and default parameters. The four gene sequences were then concatenated to produce a multilocus alignment, before poorly aligned and gapped sites were removed using trimAL [36]. A Maximum-Likelihood approach testing alternative models to determine the best combination of evolutionary models and rates was undertaken in MEGA6 for both the 16S rDNA and concatenated gene datasets. The models considered to describe the substitution pattern of these datasets the best were those with the lowest Bayesian Information Criterion (BIC) score. The General Time Reversible Model (GTR) using the Gamma distributed plus invariant sites (G+I), with 1000 bootstrap replications was used for molecular phylogenetic analysis of the multigene data. The phylogram generated from this approach was rooted using an outgroup, *Vagococcus penaei* strain CD276 (see S1 Table for a list of strains used in these analyses and their GenBank accession numbers), to produce a topology consistent with known the phylogenetic relationships of the *Enterococcus* genus based on genome-wide data as published by Lebreton et al. 2017 [37]. In addition to the above four genes, the complete 16S rDNA sequence (GenBank MF164159) was identified and selected from the annotated *Enterococcus* contig list. The complete 16S rRNA gene sequence data of the references used in the concatenated multi-locus phylogeny were also downloaded from the NCBI database. Nucleotide sequence alignments were completed in MEGA6, as before, except without alignment trimming. Phylogenetic analysis of this data set was performed employing the Hasegawa-Kishino-Yano model (HKY) using the Gamma distributed plus invariant sites (G+I), with 1000 bootstrap replications. The phylogram generated from this approach was rooted as before, using *Vagococcus penaei* strain CD276 as an outgroup.

#### Diversity profile

A microbial diversity profile of extracted and purified 16S rDNA was performed at the Australian Genome Research Facility (Brisbane, QLD, Australia) to show that the mass of organisms in tissues was *Enterococcus*, and demonstrate that the causative agent characterised in the index case was in fact present in multiple animals. The sample submitted for analysis was from the maxillary region (n = 1) of an ethanol-fixed specimen (*G*. *mutilata*) with characteristic facial and histological lesions, collected from Christmas Island during June 2016. The sample was sequenced using real-time analysis software with the Illumina MiSeq platform incorporating Nextera XT v2 Indices and Paired End Sequencing chemistry, with primers 341F (5’-CCTAYGGGRBGCASCAG-3’) and 806R (5’- GGACTACNNGGGTATCTAAT-3’). The sample generated 300 bp paired-end reads with 0.07 Gb data yield of FastQ output and Operational Taxonomic Units (OTUs) for determination of the diversity profile. Paired-end reads were assembled by aligning the forward and reverse reads using PEAR1 (version 0.9.5) [38].

## Results

### History of the outbreak and signs

**Fig 2:**
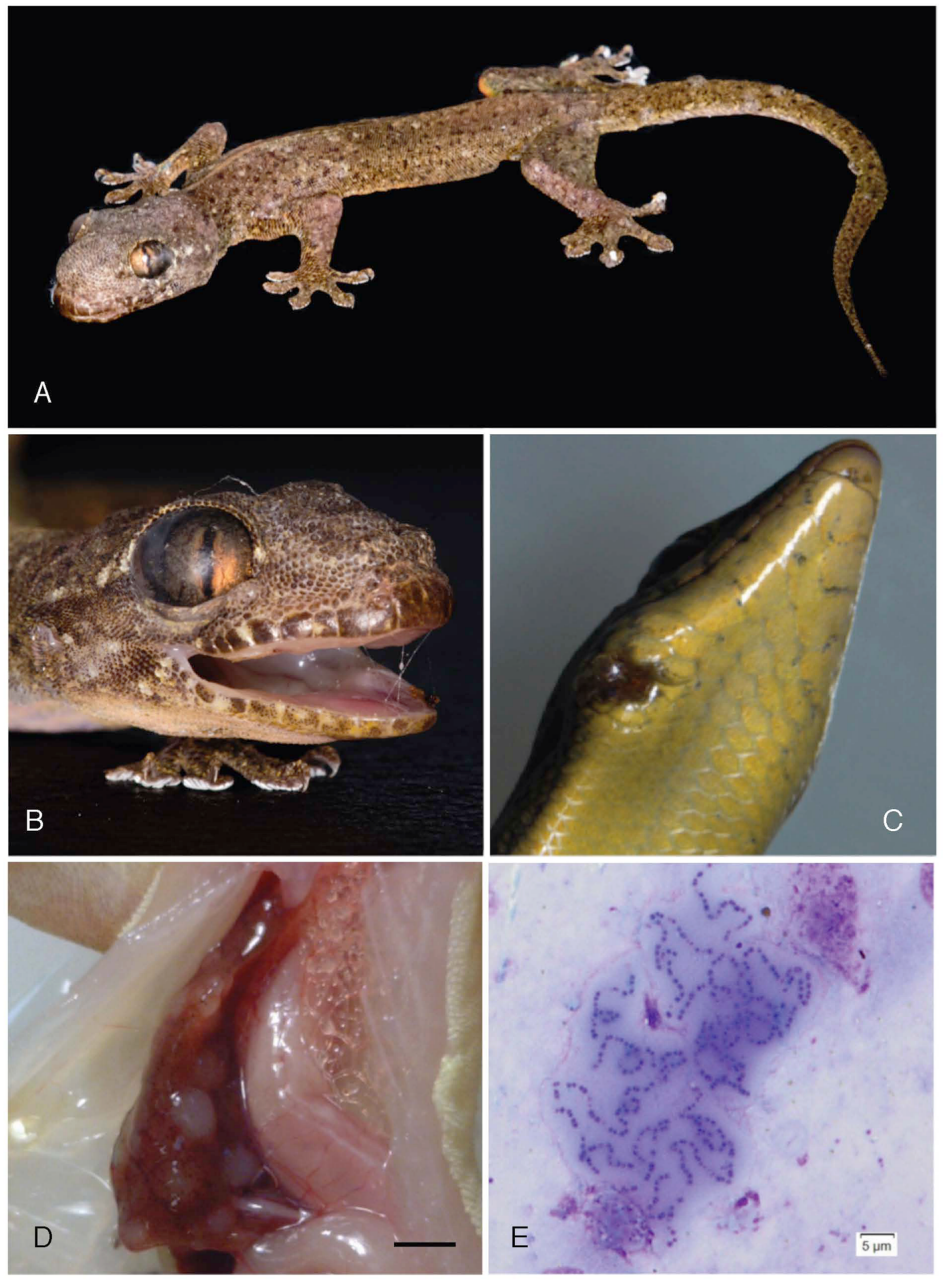
Gross and cytological findings. Gross and cytological findings in Christmas Island reptiles infected with the *Enterococcus* species bacterium included emaciation, subcutaneous nodules along the face and tail, and sub-spectacular fluid accumulation (A), sub-spectacular fluid build-up, gingival swelling and subcutaneous nodules along the face of an Asian house gecko (B), a focal skin ulcer in a blue-tailed skink (C) multiple raised white foci throughout the hepatic parenchyma of an Asian house gecko, bar 5 mm (D) and chains of cocci with a mucinous matrix aspirated from the sub-spectacular fluid of a Lister’s gecko, Romanowsky stain (E).

Beginning in October 2014, Christmas Island National Park scientific and natural resource management staff observed native and non-native reptiles exhibiting facial deformities characterised by fluid accumulation in the sub-spectacular space, irregular swellings around the mouth, gingiva and soft tissues of the head and, less frequently, subcutaneous nodules on the body or tail (Fig 2). The index case was a critically endangered Lister’s gecko held in the captive breeding colony on Christmas Island. All affected Lister’s geckos were housed in two adjacent terrariums. Affected Lister’s geckos and their cage-mates were quarantined for a period of 8.5 months, which prevented disease spread through the remainder of the captive population. Of the 47 quarantined animals, 32 succumbed to the infection, 4 were euthanased due to infection and 11 did not develop disease. Treatment was not provided to these geckos during this period. Progression of the disease was highly variable, with animals able to persist with visible lesions for a period ranging between a few weeks to four months.

A single captive blue-tailed skink from an open-air enclosure was euthanased as a precaution when it presented with an open wound on its lower right jaw (Fig 2). Histologically it was shown to be infected with the unusual bacterium; to date no further cases of infection in blue-tailed skinks have been observed. Lesions similar to those observed in the Lister’s geckos were also observed in wild invasive four-clawed geckos and Asian house geckos found at three locations on the island (Fig 1A).

### Microscopic studies

Microscopic examination of Romanowsky stained impression smears from the lesions of eight infected geckos revealed small, slightly refractile cocci occurring in chains, and less frequently as diplococci (Fig 2). These organisms were surrounded by a thick, lightly staining matrix. The organisms themselves were PAS and Gram positive, but the matrix failed to stain.

**Fig 3:**
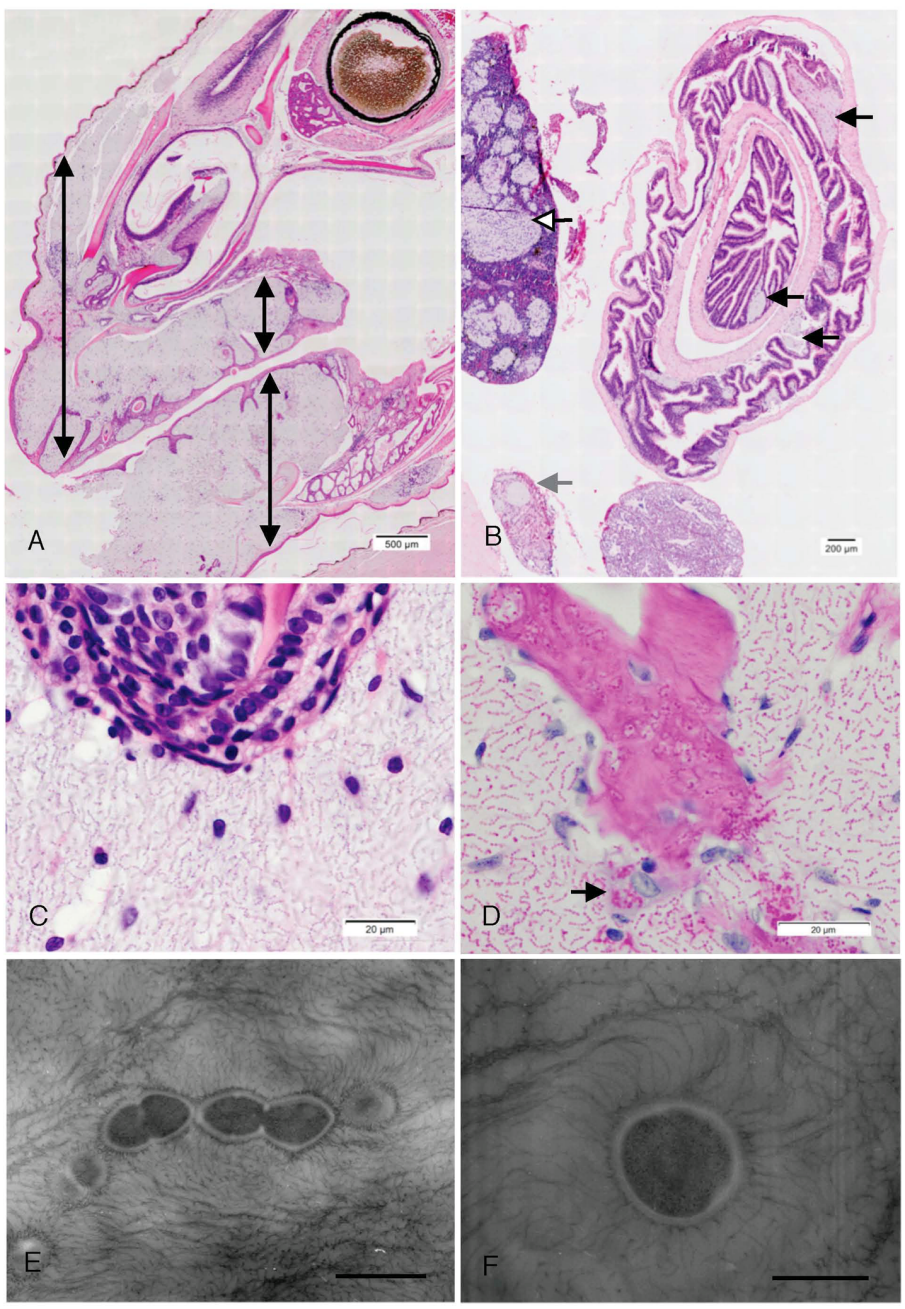
Histopathology and ultrastructure. Histological findings in an affected Lister’s gecko include cocci forming chains within a lightly staining matrix markedly distending soft tissues of the head (between double pointed arrows), H&E (A), pools of organisms within the spleen (open arrow highlights the largest aggregate), small intestinal lamina propria (black arrows), and within the parenchyma of the kidney (grey arrow), H&E (B), chains of organisms within a lightly staining matrix surrounding a germinal tooth, H&E (C), chains of organisms within a lightly staining matrix, within necrotic mandibular bone and within the cytoplasm of an osteoclast (arrow), PAS (D), electron micrographs of organisms in the liver of a four-clawed gecko illustrating cocci and diplococci, bar 500 nm (E) and a coccus with numerous pili radiating from the cell wall, bar 500 nm (F).

Microscopic examination of H&E stained tissue sections from all specimens collected revealed vast pools of organisms replacing tissues in many organs throughout the body, although, the most severe lesions were in the rostral subcutaneous and periodontal tissues (Fig 3). The inflammatory response surrounding the organisms was non-existent or mild, consisting of either a rim of mononuclear cells or an admixture of scattered granulocytes and mononuclear cells. Rare phagocytic cells, within and surrounding the pools of organisms, contained intracellular bacteria. Small clusters of extracellular organisms were evident within small lymphatic or blood vessels in the affected tissues. Organisms multifocally invaded and lysed facial bones, particularly periodontal bone. In advanced cases, organisms could be found replacing up to 30–60% of the viscera (lung, liver, spleen, intestinal wall, testes, and kidney). Histologically, the organisms were also PAS, Gram and Giemsa positive and the extracellular matrix did not take up PAS, Gram, mucicarmine or alcian blue stains. Other bacterial morphologies and protozoa were not evident within the lesions in these stained sections. Cytological and histological findings are summarised in Table 2.

**Table 2:** Summary of cytological and histological findings in infected reptiles.

Species*	Sex Age†	Presentation	Cytology	Histological Findings‡									
				Oral	Head soft tissue	Lung	Liver	Gastro-intestinal	Kidney	Skeletal muscle	Bone	CNS, eye	Other
*Ll* (1)	Ad M	Euthanasia, severe subcutaneous swellings along the head.	NA	4	4	4	4	3	4	4	4	1	The causative agent displaces 30–60% of many organs
*Ll* (1)	Unk	Live, subspectacular fluid accumulation	Pools of lightly basophilic matrix containing chains of gram and PAS positive cocci to diplococci.	NA	NA	NA	NA	NA	NA	NA	NA	NA	
*Ll* (1)	Unk	Live, subspectacular fluid accumulation	Thick basophilic matrix containing chains of small PAS and Gram positive cocci to coccobacilli. Rare yeast and hyphae.	NA	NA	NA	NA	NA	NA	NA	NA	NA	
*Ll* (2)	Ad F	Subspectacular fluid accumulation, gingivitis	Mixed cocci and long, fine bacilli. No evidence of chains of PAS positive cocci to diplococci.	4	4	4	3	3	3	3	4	3	The causative agent displaces 10–20% of many organs. Periodontal and maxillary bone lysis. Ovary, oviduct, thyroid gland, myocardium, salivary gland also affected.
*Ll* (1)	Ad F	Facial distortion. Subspectacular fluid accumulation.	NA	4	4	3	3	3	3	3	4	0	The causative agent displaces 5–20% of many organs. Severe invasion and lysis of maxilla and mandibles.
*Ll* (1)	Ad M	Euthanasia due to gingival and facial subcutaneous distension.	NA	4	4	4	3	3	3	3	4	4	The causative agent displaces 10–20% of many organs. Thyroid gland, trachea, endocardium, salivary gland also affected.
*Gm* (1)	Ad M	Euthanasia due to facial distortion.	Thick mucinous matrix containing chains of slightly refractile cocci to coccobacilli. Small numbers of erythrocytes and squamous epithelial cells.	4	4	4	4	4	4	4	4	3	The causative agent displaces 40–60% of many organs. Thyroid gland, trachea, endocardium, salivary gland also affected.
*Hf* (1)	Ad M	Facial distortion. Facial smear collected prior to euthanasia.	Thick mucinous matrix containing chains of slightly refractile cocci to coccobacilli. Rare squamous epithelial cells.	4	4	3	4	3	3	3	4	3	The causative agent displaces 5–20% of many organs.
*Gm* (1)	Ad Unk	Facial distortion.	Thick mucinous matrix containing chains of slightly refractile cocci to coccobacilli. Small numbers of erythrocytes and squamous epithelial cells.	NA	NA	NA	NA	NA	NA	NA	NA	NA	
*Ll* (1)	Ad F	Facial distortion and subspectacular fluid accumulation	Thick mucinous matrix containing chains of slightly refractile cocci to coccobacilli. Small numbers of erythrocytes, heterophils, macrophages	NA	NA	NA	NA	NA	NA	NA	NA	NA	
*Ll* (1)	SAd M	Euthanasia. Severe facial distortion.	NA	4	4	4	4	4	4	4	4	3	The causative agent displaces 20–60% of many organs.
*Ll* (1)	Ad F	Found dead. Buphthalmos. Thickened gingiva.	NA	4	4	3	3	2	1	2	2	2	The causative agent displaces 20–40% of many organs.
*Gm* (1)	Ad Unk	Euthanased. Facial distortion. Eye proptosed.	NA	4	4	2	3	1	NA	2	2	2	The causative agent displaces 40–60% of many organs.
*Ce* (1)	Ad M	Euthanased. Focal ulcer, mandible.	NA	0	2	0	0	2	2	2	0	0	Smaller, more focal aggregates of organisms often surrounded by a rim of mononuclear cells, heterophils and connective tissue.

*Species: Lister’s gecko, *Lepidodactylus listeri* (*Ll*), Four-clawed gecko, *Gehyra mutilata* (*Gm*), Asian house gecko, *Hemidactylus frenatus* (*Hf*), Blue-tail skink, *Cryptoblepharus egeriae* (*Ce*)

The number of cases for each species are shown in brackets next to the species name.

^†^Age: Adult (Ad), Sub-Adult (Sad), Unknown (Unk). Sex: Male (M), Female (F), Unknown sex (Unk)

^‡^NA = Not analysed, 0 = no lesions, 1 = small focal, 2 = large focal, 3 = multifocal, 4 = multifocally extensive pools of organisms

### Electron microscopy

Electron microscopy of negative stained ultrathin formalin-fixed tissues confirmed that the organisms were bacteria with coccoid and diplococcoid morphology. Cells possessed an electron-dense cell wall and fimbriae or pili were observed extending from the cell surface and beyond into the surrounding matrix (Fig 3). No other organisms were evident within the lesions.

### Microbiology

The only organisms that grew in any of these culture attempts were catalase positive cocci, presumed *Staphylococcus* spp. in very low density within a small number of samples from individual animals. Organisms were not seen in smears of liver or CAMs or fixed liver and CAM tissue of samples collected from the embryos. A single inoculated viper cell culture became cloudy nearing the end of the incubation period. The bacteria present in the culture were Gram positive cocci, but were catalase positive. *Enterococcus* spp. are catalase negative, therefore, the bacteria that grew in media and cell culture were considered to be contaminants or commensal organisms.

### Phylogenetic analysis of concatenated genes

**Fig 4:**
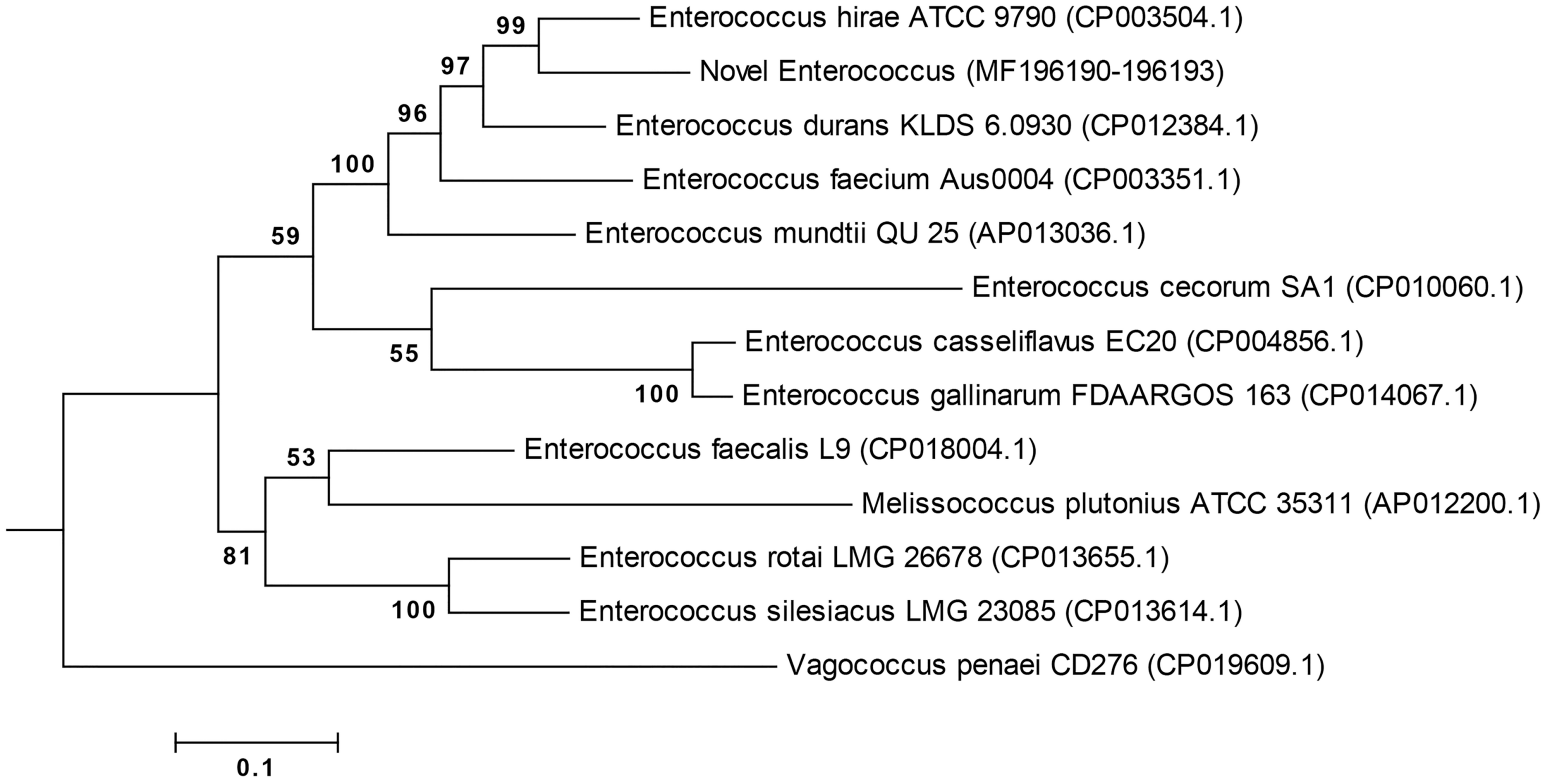
Multi-gene phylogenetic tree of concatenated genes atpA, gdh, gyd and pstC. The evolutionary history illustrated was inferred by using the Maximum Likelihood method, employing the General Time Reversible model with Gamma distributed plus invariant sites (GTR+G+I), with 13 nucleotide sequences including the novel *Enterococcus*. The percentage of trees in which the associated taxa clustered together is shown next to the branches, and is derived from 1000 bootstrap replicates. There were a total of 4082 positions in the final dataset.

The multi-locus phylogeny generated using the four concatenated genes illustrates that the ‘Novel *Enterococcus’* maps to the *E*. *faecium* species group as described by Zhang et al. [39], and is a sister species to *Enterococcus hirae* ATCC 9790 (CP003504.1) (Fig 4, with nucleotide alignment illustrated in S1 Fig). The maximum sequence similarity of the ‘Novel *Enterococcus*’ sequence to the 12 reference sequences at the genes atpA, gdh, gyd and pstC were 92%, 96%, 85% and 96% respectively.

### Phylogenetic analysis of 16S rDNA

**Fig 5:**
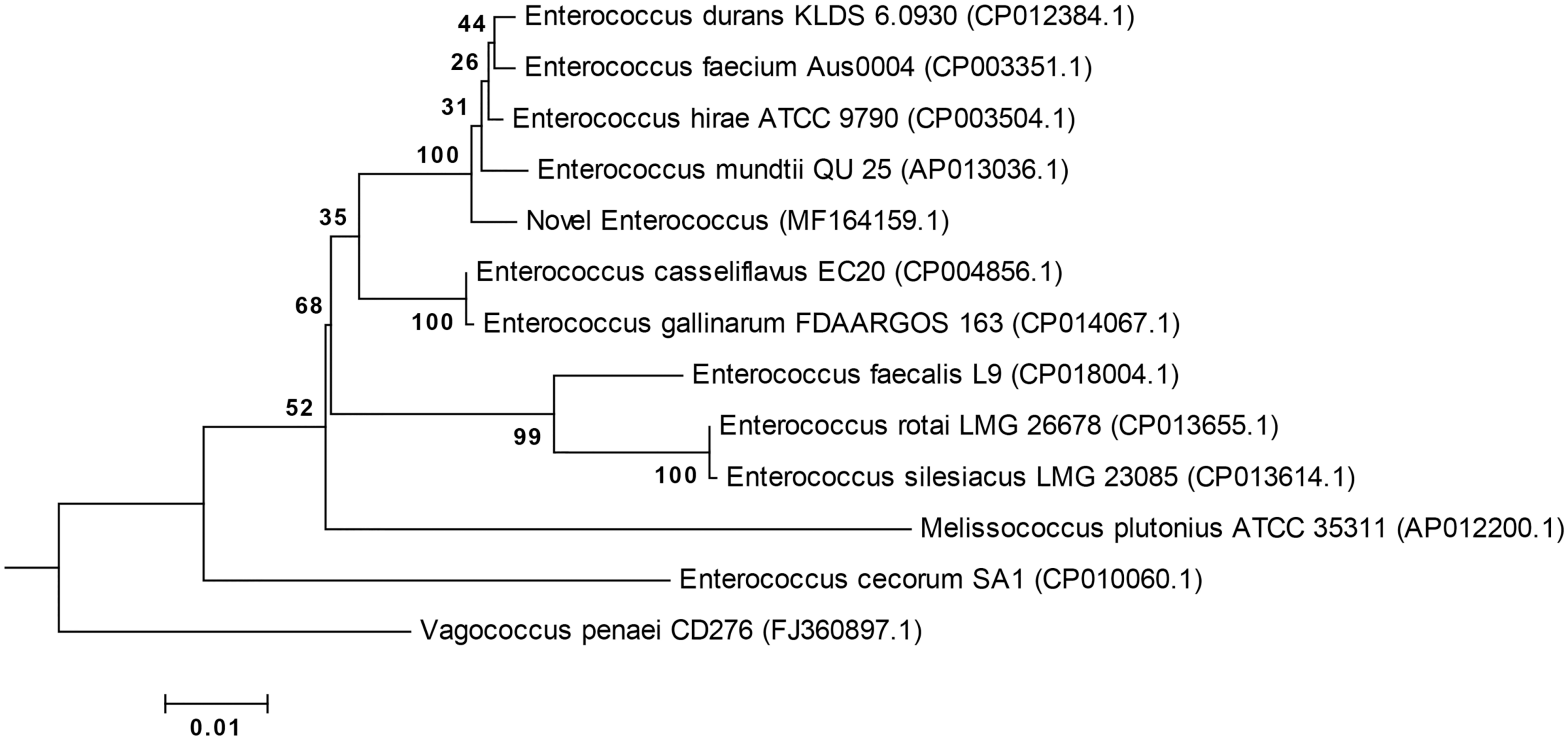
16S rDNA phylogenetic tree. The evolutionary history was inferred by using the Maximum Likelihood method, employing the Hasegawa-Kishino-Yano model with Gamma distributed plus invariant sites (HKY+G+I), with 13 nucleotide sequences including the novel *Enterococcus*. The percentage of trees in which the associated taxa clustered together is shown next to the branches, and is derived from 1000 bootstraps. There were a total of 1544 positions in the final dataset.

The newly derived sequence amplified using the panbacterial primers was unique, and showed a sequence similarity of 99% to an array of species within the *Enterococcus* genus of bacteria when blasted on the NCBI database. A phylogram created using the entire 16S rDNA sequence obtained from whole genome sequencing shows that this bacterium is an *Enterococcus* sp. (Fig 5, with nucleotide alignment illustrated in S2 Fig), however, its relationship to other members of the *Enterococcus* genus could not be defined due to poor bootstrap resolution. The maximum sequence identity of the complete 16S rDNA sequence to the 12 reference sequences included in the phylogram was 99%.

### Diversity profile

**Fig 6:**
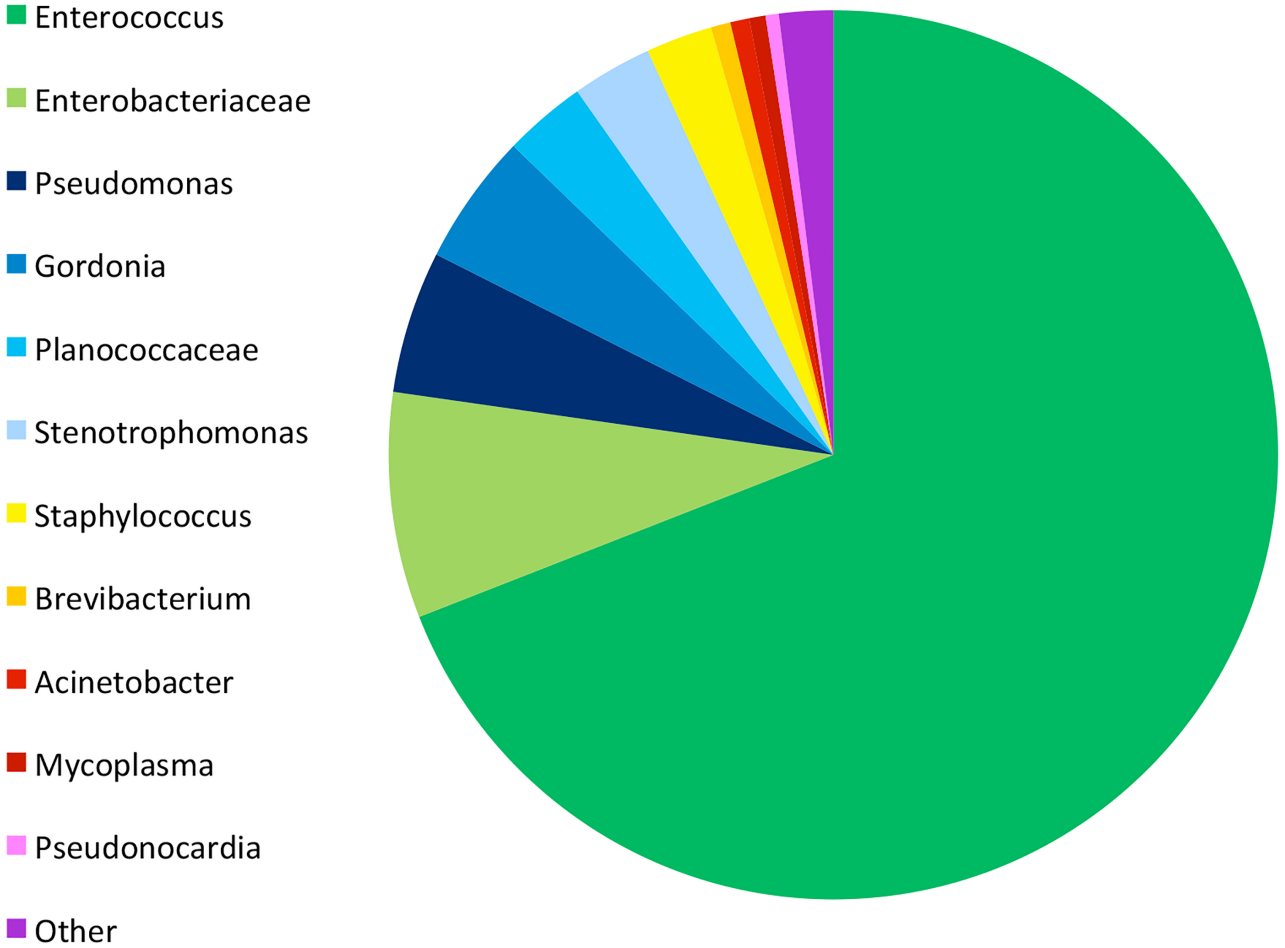
Diversity profile. A diversity profile obtained from sequencing a maxillary lesion from a *H*. *frenatus* specimen. The relative abundance and proportions of various bacteria are represented.

A microbial diversity profile (Fig 6) was performed on a maxillary lesion from a *H*. *frenatus* specimen that was confirmed to be infected by means of gross inspection, histopathological examination and qPCR assay. The majority of bacteria detected in the sample was from the *Enterococcus* genus (69.02%). When compared to the NCBI database, this sequence showed 100% homology to an array of enterococcal species. Other significant proportions of bacterial microorganisms existing in the mixed microbial community included *Enterobacteriaceae* (8.2%), *Pseudomonas* (5.17%), and *Gordonia* (4.78%). The remaining sequences were those expected for commensal bacteria present in the oral microbiome.

### Retrospective analysis

Examination of Christmas Island reptiles from the Australian Museum collection, reptiles collected as part of an island-wide health disease survey, and reptiles that died spontaneously in the Taronga Zoo captive breeding program failed to find grossly or microscopically similar lesions to those seen in the infected lizards. Thus, the infection seems to have recently emerged and spread rapidly across species and island terrain. Culture of pooled reptile tissues collected on Christmas Island in 2010 did not identify any evidence of viral pathogens.

## Discussion

This investigation has resulted in the identification of a bacterial infection of unusual morphology in captive endemic and free-ranging invasive reptiles on Christmas Island. Based on the staining characteristics, ultrastructure, and phylogenetic analysis, the etiological agent is a novel *Enterococcus* species. The syndrome associated with this infection includes chronic, progressive and invariably fatal illness characterised by weight loss, slow movement, and swellings of facial soft tissues. Multisystemic pools of the organism were most severe in tissues surrounding the teeth and within the mandibles, associated with a minimal to non-existent host inflammatory response.

There is evidence that this is not an isolated incident, and outbreaks, possibly caused by this or a similar organism have occurred in other parts of the world, in other species of reptiles. A morphologically identical syndrome is described in two Singapore house geckos (*Gekko monarchus*) and is ascribed to a *Streptococcus* species [40]. At the time the publication was written *Enterococcus* species were classified within the genus *Streptococcus*, so it is possible that the pathogen was actually an *Enterococcus* species. Additional similar cases are described by Zwart and Cornelisse, 1972 [41] where a presumed *Streptococcus* species with a thick “capsule” is identified in chronic, progressive and fatal infections in Carolina anole (*Anolis carolinensis*), Cape girdled lizards (*Cordylus cordylus*), Balkan green lizards (*Lacerta trilineata*), and European green lizards (*Lacerta viridis*). Attempts to culture the organism were unsuccessful. The authors went on to experimentally inoculate 13 common wall lizards (*Podareis muralis*) that subsequently developed chronic, fatal infections [41]. These previous events seem identical to those in Christmas Island reptiles, except that they were identified only in captive animals, the organism was eradicated through quarantine, and the organism in the Singapore house geckos grew in culture. Unfortunately, case material from these outbreaks was unavailable and additional comparisons could not be made.

Despite numerous attempts with varied media and conditions, the bacterium from Christmas Island reptiles did not grow in culture. Various *Enterococcus* spp. require selective media for isolation and culture [30]. Specific selective agents, growth factors, especially B-group vitamins, and growth conditions are necessary as enterococci are fastidious organisms, differing from most other Gram positive bacteria [31]. Therefore, it is possible that the correct medium for the growth of this *Enterococcus* is yet to be determined. Furthermore, despite the numerous attempts, culture may have been unsuccessful as a result of the bacterial cells dying or being rendered non-viable from the freezing, preservation or storage methods employed. The delay between collections and culture attempts during transport from Christmas Island to Australia may have also damaged the integrity of the organism, contributing to a lack of observed growth on media.

The organism causing this disease was demonstrated to be an *Enterococcus* species, initially, by comparing a small fragment of the 16S rDNA amplified using panbacterial primers to *Enterococcus* sequences. The precise relationship between this and other *Enterococcus* spp. was not resolved when the entire 16S rDNA sequences were compared. This is consistent with the findings of previous authors who found that 16S rDNA analysis offers resolution for differentiation between the main groups of enterococci, however fails to discriminate closely related enterococcal species [34, 39, 42, 43].

To gain a more discriminatory characterisation of the causative agent compared with known *Enterococcus* species, a multi-locus phylogeny involving the concatenation of constitutive protein-coding genes from four loci (atpA, gdh, gyd and pstC), resolved by whole genome sequencing was created. Based on these findings, and relative divergence from known species, we conclude that the organism found in the lizards from Christmas Island represents a novel *Enterococcus* species. Future investigations using DNA obtained from cultured organisms or infected tissue, where the bacterial DNA has been enriched through removal of CpG methylated eukaryotic DNA, and supplementary whole genome sequencing, will allow a better understanding of the pathogenicity and metabolism of this organism [44].

Enterococci are Gram positive, non-spore forming coccoid or diplococcoid organisms belonging to the lactic acid bacteria. These organisms are tolerant of a broad range of environmental conditions including environmental oxygen concentrations, temperature, pH and salt concentrations that would often be considered extreme for other bacteria [17, 23, 45]. While many species of enterococci are commensal within the alimentary tract [17, 19, 20, 45–48], organisms in this genus are fast-emerging as human [17, 21, 45] and reptile pathogens [48–51].

Enterococci in humans and in reptiles are known for their high prevalence of multidrug resistance [17, 19, 21, 45]. In particular, *E*. *faecium* has emerged as a significant opportunistic animal pathogen [52], and currently represents more than 90% of *Enterococcus* isolates in humans and animals [53]. Enterococci have numerous virulence factors, including the potential to form extracellular polymeric substances (EPS), which assist in resistance to phagocytosis, and the capacity to form biofilms [54, 55]. In the genus *Enterococcus*, the formation of pili is an essential element for biofilm production and an important factor in the pathogenesis of human endodontic, urinary tract, and endocardial infections [21]. The Christmas Island reptile bacterium appears to potentially represent an extreme example of biofilm production. The pili radiating from the cell wall most likely contribute to the organism’s ability to form biofilms that expand within animal tissues. The formation of biofilms by this organism across a range of tissues is of concern because it seems to be protecting the organism from the host’s immune response. This is consistent with the finding that the EPS of the *E*. *faecalis* biofilm masks the lipoteichoic acids of its cell wall from detection by agglutinating antibodies. Additionally, biofilm production renders bacterial pathogens difficult to eradicate, and they are a predictor of chronic infection [55] as a result of the following factors: biofilm producing bacteria are up to 1000 times more resistant to phagocytosis, and are more resilient to antibiotic treatment, biofilms allow organisms to persist without dividing to further evade many antibiotics, biofilms support bacterial growth by controlling nutrient and microenvironment, pili associated with biofilms assist with tissue adhesion, and biofilms can aid microbial gene transfer facilitating the spread antimicrobial resistance [54, 56–60].

The source of the infection in the captive breeding facility of Lister’s geckos and blue-tailed skinks is unknown. Based on the geographic distribution of the disease in wild reptiles, the organism appears to be present either in the environment or in a living reservoir across a significant portion of Christmas Island. Wild insects used for food, and soil and vegetation used in the enclosures are potential sources of infection, but would seem unlikely as outbreaks only occurred in two adjacent terrariums in the indoor breeding facility and one enclosure in the outdoor breeding facility. Alternately, exposure to free-ranging feral reptiles that routinely enter the breeding facilities could have been the infection source. Control of the bacterial infection in Lister’s geckos and blue-tailed skinks on Christmas Island has been achieved in the short-term through quarantine of infected animals. Given the presence of infected invasive species free-ranging within the breeding colony and food production facility, additional infections over time seem likely.

## Conclusions

Conservation management actions for both the Lister’s gecko and blue-tailed skink are potentially impacted by this emergent pathogen, including captive breeding, potential reptile translocations to other islands, and housing reptiles in larger outdoor enclosures. Due to the unusual nature of the organism and the emergence of disease through the captive and free-ranging lizards on the island, further investigation is required to characterise the disease by developing a comprehensive understanding of the ecology of the organism. Additional surveillance of the giant gecko population is being conducted and it may be wise to consider a captive breeding group of these animals on mainland Australia in the event that this disease is identified in that species. A disease risk analysis could be conducted to unite stakeholders, to guide conservation management of the two critically endangered species, and to determine what biosecurity measures are warranted to further protect reptile biodiversity on Christmas Island, the Cocos (Keeling) Islands, and mainland Australia [61].

Further analysis of whole genome sequence data is being pursued to identify virulence factors, antibiotic resistance, and facilitate the development of a species-specific PCR. A species-specific diagnostic tool is necessary for more extensive epidemiological studies and effective management action. Additional research to further investigate environmental conditions supporting bacterial growth, zoonotic potential, and drug resistance patterns of this organism would also be highly valuable.

## Supporting information

S1 Table Bacterial strain and GenBank accession numbers included in phylogenetic analyses. (DOC)

S1 Fig Nucleotide alignment resulting from multi-gene comparison of concatenated genes atpA, gdh, gyd and pstC. The nucleotide alignment of 13 bacteria, including the novel *Enterococcus* and *Vagococcus penaei* used to root the phylogenetic tree, was made for each of the genes (atpA, gdh, gyd, pstC) with MEGA6 using the CLUSTALW alignment method and default parameters.
(PDF)

S2 Fig Nucleotide alignment resulting from 16S rDNA analysis. The nucleotide alignment of 13 bacteria, including the novel *Enterococcus* and *Vagococcus penaei* used to root the phylogenetic tree, was made to represent the16S rDNA analysis with MEGA6 using the CLUSTALW alignment method and default parameters.
(PDF)
