# Supplementary material for: Emergent multisystemic Enterococcus infection threatens endangered Christmas Island reptile populations
Source: PLoS One. 2017 Jul 20;12(7):e0181240. doi: 10.1371/journal.pone.0181240 (PMC5519069; doi:10.1371/journal.pone.0181240)
Supplement: S1 Table — (DOC) [file pone.0181240.s001.doc]

**S1 Supplementary Table 1: Bacterial strain and GenBank accession numbers included in phylogenetic analyses.**

| **Sequence species** | **Strain no.** | **GenBank Accession no.** |
| --- | --- | --- |
| Novel *Enterococcus* atpA | From uncultured reptile tissue | MF196190 |
| Novel *Enterococcus* gdh | From uncultured reptile tissue | MF196191 |
| Novel *Enterococcus* gyd | From uncultured reptile tissue | MF196192 |
| Novel *Enterococcus* pstC | From uncultured reptile tissue | MF196193 |
| Novel *Enterococcus* 16S rDNA | From uncultured reptile tissue | MF164159 |
| *Enterococcus faecium* | Strain Aus0004 | CP003351.1 |
| *Enterococcus hirae* | Strain ATCC9790 | CP003504.1 |
| *Enterococcus durans* | Strain KLDS 6.0930 | CP012384.1 |
| *Enterococcus mundtii* | Strain QU 25 | AP013036.1 |
| *Enterococcus cecorum* | Strain SA1 | CP010060.1 |
| *Enterococcus casseliflavus* | Strain EC20 | CP004856.1 |
| *Enterococcus gallinarum* | Strain FDAARGOS 163 | CP014067.1 |
| *Enterococcus faecalis* | Strain L9 | CP018004.1 |
| *Melisococcus plutonius* | ATCC 35311 | AP012200.1 |
| *Enterococcus rotai* | Strain LMG 26678 | CP013655.1 |
| *Enterococcus silesiacus* | Strain LMG 23085 | CP013614.1 |
| *Vagococcus penaei* | Strain CD276 | CP019609.1 |
