# Supplementary material for: Emergent multisystemic Enterococcus infection threatens endangered Christmas Island reptile populations
Source: PLoS One. 2017 Jul 20;12(7):e0181240. doi: 10.1371/journal.pone.0181240 (PMC5519069; doi:10.1371/journal.pone.0181240)

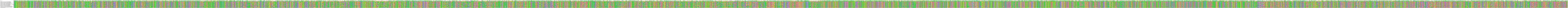

Supplement: S1 Fig — The nucleotide alignment of 13 bacteria, including the novel Enterococcus and Vagococcus penaei used to root the phylogenetic tree, was made for each of the genes (atpA, gdh, gyd, pstC) with MEGA6 using the CLUSTALW alignment method and default parameters. (PDF) [file pone.0181240.s002.pdf]
