# Supplementary material for: Emergent multisystemic Enterococcus infection threatens endangered Christmas Island reptile populations
Source: PLoS One. 2017 Jul 20;12(7):e0181240. doi: 10.1371/journal.pone.0181240 (PMC5519069; doi:10.1371/journal.pone.0181240)
Supplement: S2 Fig — The nucleotide alignment of 13 bacteria, including the novel Enterococcus and Vagococcus penaei used to root the phylogenetic tree, was made to represent the16S rDNA analysis with MEGA6 using the CLUSTALW alignment method and default parameters. (PDF) [file pone.0181240.s003.pdf]

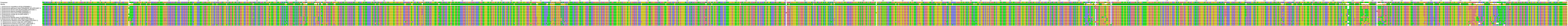
Consensus
Identity
1. Enterococcus casseliflavus EC20 (CP004856.1)
2. Enterococcus gallinarum strain FDAARGOS 163 (CP014067.1)
3. Enterococcus durans strain KLDS 6.0930 (CP012384.1)
4. Enterococcus faecium Aus0004 (CP003351.1)
5. Enterococcus hirae ATCC 9790 (CP003504.1)
6. Enterococcus mundtii QU 25 (AP013036.1)
7. Novel Enterococcus
8. Enterococcus faecalis strain L9 (CP018004.1)
9. Enterococcus rotai strain LMG 26678 (CP013655.1)
10. Enterococcus silesiacus strain LMG 23085 (CP013614.1)
11. Enterococcus cecorum strain SA1 (CP010060.1)
12. Melissococcus plutonius ATCC 35311 (AP012200.1)
13. Vagococcus penaei strain CD276 (FJ360897.1)
